# Small molecule KDM4s inhibitors as anti-cancer agents

**DOI:** 10.1080/14756366.2018.1455676

**Published:** 2018-04-13

**Authors:** Hongzhi Lin, Qihang Li, Qi Li, Jie Zhu, Kai Gu, Xueyang Jiang, Qianqian Hu, Feng Feng, Wei Qu, Yao Chen, Haopeng Sun

**Affiliations:** a Department of Medicinal Chemistry, China Pharmaceutical University, Nanjing, China;; b Department of Natural Medicinal Chemistry, China Pharmaceutical University, Nanjing, China;; c School of Pharmacy, Nanjing University of Chinese Medicine, Nanjing, China

**Keywords:** Cancer, epigenetic, histone demethylation, KDM4s inhibitors

## Abstract

Histone demethylation is a vital process in epigenetic regulation of gene expression. A number of histone demethylases are present to control the methylated states of histone. Among these enzymes, KDM4s are one subfamily of JmjC KDMs and play important roles in both normal and cancer cells. The discovery of KDM4s inhibitors is a potential therapeutic strategy against different diseases including cancer. Here, we summarize the development of KDM4s inhibitors and some related pharmaceutical information to provide an update of recent progress in KDM4s inhibitors.

## Introduction

Post-transcriptional modifications on histones are vital for cells to regulate the expression of genes in epigenetic level. Through affecting micro-environment or recruiting functional proteins, the modifications play different roles in cells. There are several types of histone modifications: acetylation, methylation, phosphorylation, ubiquitylation, sumoylation, etc.^1–3^. Acetylation and methylation are most significant and well-studied histone modifications among them. Successes on histone deacetylase (HDAC) inhibitors in the pharmaceutical area have stimulated the development of histone methylation regulatory agents, including histone lysine demethylase (KDM) inhibitors^4–7^. Like HDAC, some KDMs are thought as new targets for anti-cancer therapy. Comparing with acetylation at lysine residues on histone tails can neutralize the positive charge of histones to promote transcription, the methylation on lysine side chains does not lead to a charge shift^8^. The functions of histone methylation are more complex and context-dependent^8^. The methylated marks (on different positions and states) need to be read by some downstream proteins or protein complexes (such as HDACs), which can regulate the genetic transcription or some other biological processes. Therefore, different histone methylation marks have different functions (transcriptional activation or suppression, and some other functions)^9–11^. Moreover, the huge number of histone modification proteins also provides some confusion about the regulation of histone modification. So far, more than 30 methyltransferases and 20 demethylases have been identified, and the numbers are still increasing^11^^,^^12^. These enzymes methylate and demethylate different sites and states of histone methylated marks. But, the cooperative or antagonistic relationships among them are hard to be elucidated completely.

So far two families of KDM have been discovered^13^^,^^14^. One is lysine-specific demethylase (LSD) that belongs to flavin adenine dinucleotide (FAD)-dependent monoamine oxidase. Two members, LSD1 and LSD2, are in this family. Most, if not all, other KDMs belong to Jumonji domain containing lysine demethylases (JmjC KDMs). More than 20 JmjC KDMs have been identified and divided into six subfamilies (KDM2–7) with different demethylated catalysis activities. Although the biological roles of each subfamily are still not elucidated completely, the KDM4s are regarded as promising targets for cancer therapy^15–17^.

KDM4A (JmjD2A/JHDM3A) and other members (KDM4B/JmjD2B/JHDM3B, KDM4C/JmjD2C/JHDM3C, and KDM4D/JmjD2D/JHDM3D) in KDM4 subfamily are earliest identified as JmjC KDMs which can catalyze the demethylation of histone H3 subunit lysine9 tri-/di-methylated mark (H3K9me_3/2_)^18^. High expressions of KDM4s are considered as promote oncogenesis in some types of cancers, including prostate^19^, breast^20^^,^^21^, colon^22^, and some others^15^. Downregulation of KDM4s via molecular biology methods or inhibition of their catalytic activity by small molecule inhibitors is confirmed as strategy for oncotherapy^16^. In recent decades, increasing attention is focused on the development of KDM4s inhibitors as antitumor agents. Meanwhile, a number of inhibitors have been disclosed by medicinal chemists. Recently, some potent and selective JmjC KDM inhibitors were reported. Although the good clinical candidates are still undiscovered, many publications have reported various modulators with different chemotypes under the guide of different design strategies or screen assays (especially high-throughput screen, HST). Although some excellent reviews have summarized the development of KDM4s inhibitors^23–29^, it is necessary to provide an update for new drug discovery and design works.

In this review, we first introduce the biological roles of KDM4s and some related JmjC KDMs. Second, we demonstrate some structural information of these proteins is summarized based on several reported protein crystal structures with or without co-factor or inhibitor. We mainly emphasize KDM4s inhibitors with different chemotypes and inhibitory mechanisms. Structure-based drug design (SBDD) strategy which is widely applied in the design of KDM4s inhibitors is highlighted. Finally, some prospects about design and development of KDM4s inhibitors are introduced.

## Biological function of KDM4s

KDM4s carry a wide range of biological functions including but not limited to removing histone methylated marks (histone code eraser). They are involved in many biological processes such as transcriptional regulation, cell cycle, senescence, DNA damage response, and heterochromatin formation. These roles are vital for cell growth and their over-expressions are crucial for some types of cancers.

All the four members of KDM4s subfamily can catalyze the demethylation H3K9me_3/2_, furthermore, the KDM4A ∼ C can also demonstrated to demethylate H3K36me_3_^30^. KDM4s have also been demonstrated to H1.4K26me_3/2_^31^. The positions and states of the methylated marks on histone mostly decide the roles of enzymes in the transcriptional regulation. H3K9me_3/2_ is a transcriptional suppressive mark that is associated with heterochromatin assembly^32^^,^^33^. The roles of H3K36me_3_ are indistinct at some level, but it is generally believed that this histone mark inhibits transcription at start but facilitates transcriptional elongation^16^^,^^34^^,^^35^. Thus, KDM4s are thought as transcriptional activators. In fact, overexpression of KDM4A indeed promotes genetic transcription in some types of cancers^21^^,^^22^^,^^36^. But the relationship between the activity roles of KDM4s and genetic transcription is not very solid: KDM4A directs the repression of E2F responsive promoters^37^. Additionally, some studies show that KDM4s catalysis is not only limited to demethylation but also to act on N-alkyl groups other than methyl^38^. Beyond the roles of catalysis, the antagonism between KDM4A and heterochromatin protein 1 gamma (HP1γ) during cell cycle also controls the chromatin accessibility and facilitates transcription^39^.

As a transcription-associated protein, KDM4A, the same as other KDM4 members, is a co-activator of some nuclear receptors mostly including androgen and oestrogen receptors (AR and ER). KDM4A, 4C, and 4D are all co-activators for AR-related genes by binding AR directly and their overexpressions stimulate the AR’s function. KDM4B also can directly bind AR to keep its stability by inhibiting its ubiquitination in prostate cancer cells^21^^,^^40–43^. The co-factor roles of KDM4s may be vital for the growth of cancer cells because siRNA-blocking of KDM4A indicates the inhibition of cancer cell growth in some cell-based experiments^44^^,^^45^. Some small molecule inhibitors can also suppress cancer growth and it indicates that the functions of KDM4A are depended on its catalytic activity. But KDM4A-siRNA-induced gene silence inhibited not only the proliferation of the ER-positive but also the ER-negative breast cancer cell lines (MCF-7 and MDA-MB-231)^44^^,^^45^. These results implied that KDM4A may also benefit the proliferation of breast cancer cells via some other mechanisms.

KDM4s are involved in some protein complexes. KDM4A directly binds HDAC and retinoblastoma (pRb) to form a complex. In this complex, KDM4A associated with pRb and class I HDACs and mediated repression (but not promotion as a transcriptional activator) of E2F-regulated promoters *in vivo*^37^. One of the E2F-regulated genes *aplasia Ras homolog member I* (*ARHI*), which is a tumour suppressive gene, is repressed by this complex^46^. The downregulation of ARHI may lead to the silence of specificity protein1 (SP1) in advanced breast cancer cells and promotes the metastasis of cancer. The transcription factor SP1 has been considered as an oncoprotein which is highly expressed in early-stage breast cancer. But in advanced breast cancer cells, SP1 is downregulated due to its inhibitory roles for migratory and invasive abilities. Moreover, SP1 also interact with KDM4A strongly which may be a reason for the SP1-inactivity. Taken together, KDM4A seems to play an important role in SP1 downregulation in advanced breast cancer cells. However, in some advanced human bladder cancers, downregulation of KDM4A may promote lymphovascular invasion^47^.

p53 is a well-known tumour suppressor. It is also a potential non-histone substrate of KDM4s. The function of p53 in senescence process is biphasic and related to KDM4s: p53 is essential in the early phase of senescence but must be removed in the late phase. In this process, the SCF^Fbxo22^–KDM4A–p53 complex is an indispensable element which functions as an E3 ubiquitin ligase complex targeting methylated p53 for its degradation at the late senescent stage. The overexpression of a catalytic mutant KDM4A^H188A^ results in p53 stability^48^. Thus, blocking the interactions between KDM4A and some other proteins (such as pRb and SP1) may be a therapeutic strategy for some types of cancers. However, SCF^Fbxo22^ complex was also identified as an E3 ubiquitin ligase for KDM4s themselves^49^. The FIST-C domains of the Fbxo22 subunit interact with JmjC and JmjN domains of KDM4A to mediate the ubiquitination of KDM4A. SCF–FbxL4 complex is another ubiquitin ligase for KDM4A^50^. A previous study has revealed that siRNA-interference of KDM4A was sufficient to trigger a p53-dependent senescence response via transcriptional negative regulating CHD5 (Chromodomain-helicase-DNA-binding protein 5), a tumour suppressor, in A549 lung cancer cells^51^.

In DNA damage response process, 53BP1 plays a crucial role and binds to methylated H4K20 at DNA damage sites^52^. KDM4A can bind to methylated H4K20 via its tudor domains. The degradation of KDM4A is necessary for the recruitment of 53BP1 to DNA damage sites. Two E3 ubiquitin ligases, RNF8 (ring finger protein 8) and RNF168, degrade KDM4A via ubiquitin–proteasome pathway to trigger DNA damage repair process^53^. Thus, the overexpression of KDM4A may interfere with the DNA damage repair.

The protein level of KDM4A changes in a cell-cycle-dependent manner and is high in G1/S phase. The overexpression of KDM4A, but not catalytical inactive KDM4A^H118A^, resulted in a faster S phase^39^. In contrast, silencing KDM4A with KDM4A–siRNA induced G2/M and S phase arrests in a LPS-stimulated p53-null neuroectodermal stem cell model^54^.

The overexpression of KDM4A also plays significant roles in some other types of cancers^15^. In colon cancer HCT116 cells, KDM4A interacted with p53 directly^22^. The downregulation of KDM4A resulted in increased expression of p21 and Puma protein, whereas the level of the anti-apoptotic Bcl-2 protein decreased. Knockdown of KDM4A led to reduction of the proliferation in three different colon cancer cell lines (HCT116, DLD-1, and HT-29). Similar role of KDM4A is also found in gastric cancer^36^. Overexpression of KDM4A in Kaposi’s sarcoma-associated herpesvirus (KSHV) increases its reactivation^55^. KDM4B is involved in neural stem cells (NSCs) inflammation. In an LSP-stimulated *in vitro* model, knockdown of KDM4B downregulated the expression levels of p65, iNOS, Bcl2, and TGF-beta^56^. This function was dependent on its H3K9me_3/2_ demethylated activity. For embryonic stem cells (ESCs), the reduction of H3K9me_3/2_ level by KDM4B improved the reprograming of ESCs into cloned embryos^57^. The same as KDM4A, KDM4B plays a significant role in cancer. Beside its ER-related functions in breast cancer^20^^,^^58^, KMD4B is highlighted in gastric cancer. KDM4B was also regulated by hypoxia and radiation to promote the proliferation in gastric cancer cell^59^. It was expressed in gastric cancer cells and was required for the proliferation and survival of tumour. Silence of KDM4B gene by siRNA inhibited cell growth and elevated the level of p53 and p21 protein^60^. Cooperating with β-catenin, KDM4B could enhance gastric cancer metastasis and colon cancer cell proliferation^61^^,^^62^. Depletion of KDM4C (which is regarded as oncogene) from cancer cells resulted in inhibition of proliferation of cancer cells^41^^,^^63^.

KDM4C is a hypoxia-inducible factor 1α (HIF-1α) co-activator that enhances the expression of GLUT1, LDHA, and other HIF-1α-related genes. It is also required for breast cancer progression^64^. The oncoprotein Mouse Double Minute 2 (MDM2) was up-regulated by high level of KDM4C, too^65^.

Compared with KDM4A-C, KDM4D encodes only a shorter protein consisting of JmjN and JmjC domain. Methylation of H3K79 has been linked to actively transcribed genes and is involved in the regulation of telomeric silencing, cellular development, cell-cycle checkpoint, DNA repair, and regulation of transcription^66^. In a recent research^67^, KDM4D was validated to affect H3K79me_3_ level *in vivo*: upregulation of KDM4D led to a severe reduction in H3K79me_3_ level in cells, despite the result *in vitro* assay revealed that KDM4D showed no reproducible reduction in H3K79me_3_. Even though, KDM4D lysine demethylase might be a potential regulator for trimethylated H3 at Lys79.

## Protein structure and demethylation mechanism of KDM4A

### Protein structure of KDM4A

The structural information of KDM4s is vital for rational drug design of inhibitors. KDM4A, 4B, and 4C share several domains: JmjN domain, JmjC domain, tandem PHD domain, tudor domain, and F-box domain. The interaction between JmjN and JmjC domain is important for the catalytic function and stability of KDM4s^68^. Both PHD domain and tudor domain can recognize the methylated lysine mark on H3/H4 tail of histone, but the function of PHD domain seems not dependent on H3 N-terminal^69^. F-box domain is involved in interaction between KDM4A and the SKP1-Cul1-F-box ubiquitin ligase^50^. KDM4C is much shorter than KDM4A and 4B: the PHD and tudor domains are not involved in its gene sequence.

**Figure 1.**
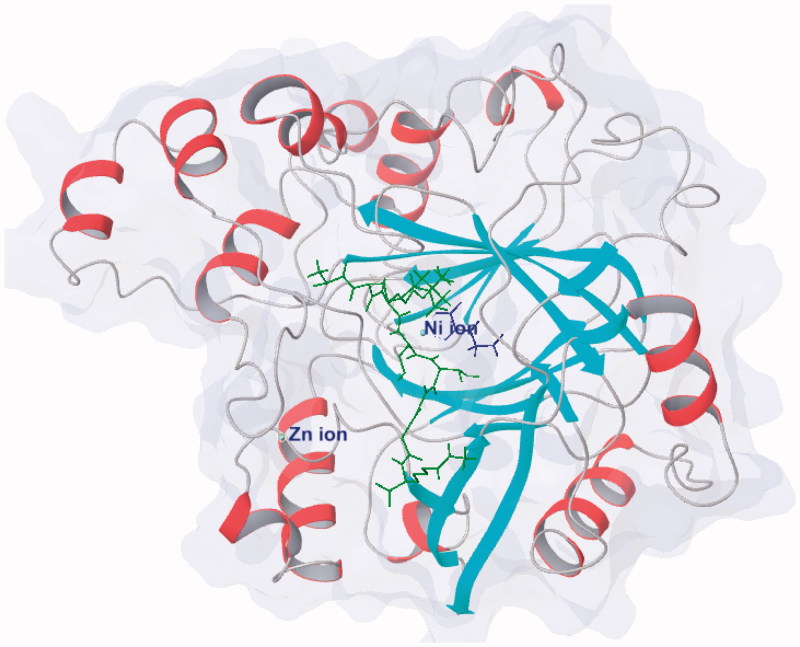
Protein structure of KDM4A (PDB code: 2OQ6) shows as white/red rainbow; H3K9me3 peptide shows in green; 2-OG shows in blue.

KDM4A and other JmjC KDMs contain a JmjC domain as catalytic region^18^^,^^70^^,^^71^. This JmjC domain is conserved in different species and belongs to the superfamily of Fe(II) ion and 2-OG dependent oxygenase (Figure 1)^63^^,^^72^. In this domain, a Zinc ion is bound by residues Cys234, His240, Cys306, and Cys308^71^. The zinc-finger motif is much close to histone substrate peptide recognition region. Two adjacent residues, Lys241 and Arg309, are involved in substrates reorganization. It indicates that the zinc-finger motif plays an important role for keeping the substrate-binding conformation. This result is in accordance with the study of KDM4A Zn-ejection inhibitors^73^.

**Figure 2.**
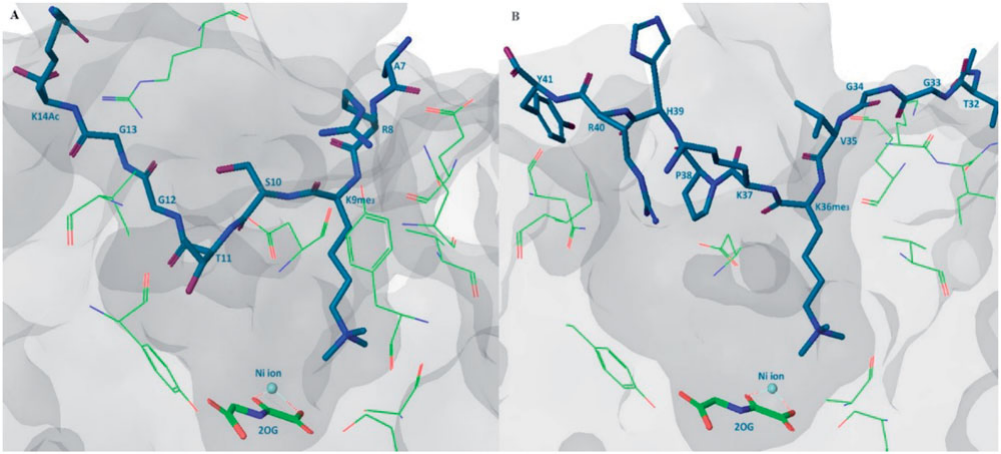
Substrate binding pose. (A) KDM4A-H3K9me_3_ peptide-2-OG structure (PDB code: 2OQ6), H3K9me_3_ peptide shows in purple stick, 2-OG shows in green stick, some important residues of KDM4A shows in green line; (B) KDM4A-H3K36me_3_ peptide-2-OG structure (PDB code: 2OS2), H3K36me_3_ peptide shows in purple stick, 2-OG shows in green stick, some important residues of KDM4A shows in green line.

KDM4s can demethylate the histone substrates in different rates^30^. H3K9me_3_ is the most preferable substrate and H3K9me_2_ along with H3K36me_3_ can also be demethylated by KDM4A but with lower catalytic rate. Additionally, some studies show that the KDM4s can also catalyze the demethylation of H3K27me_3/2_ substrate^74^. Crystal structures revealed that the binding positions of H3K9me_3_ peptide and H3K36me_3_ peptide are very superimposable. In catalytic region, H3K9me_3_ substrate binds approximately in a broad “W” shape while H3K36me_3_ substrate is in a “U” shape. The H3Ser10 side chain forms a hydrogen bond with the main-chain H3Gly12 and this intra-interaction is vital for keeping the binding conformation. Ser10Ala mutant leads to a strongly reduced activity. This result is in accordance with a previous study that the phosphorylation at H3Ser10 is a “switch” for the regulation of genetic transcription. Phosphorylation at H3Thr11 also plays a similar role. In the same way, mutants of H3Gly12 and H3Gly13 also reduce the affinity. H3Pro30Gly mutant makes H3K27me_3_ peptide substrate gain activity. This “W” or “U” conformation may be important for the design of substrate-mimic inhibitors^73^ (Figure 2).

**Figure 3.**
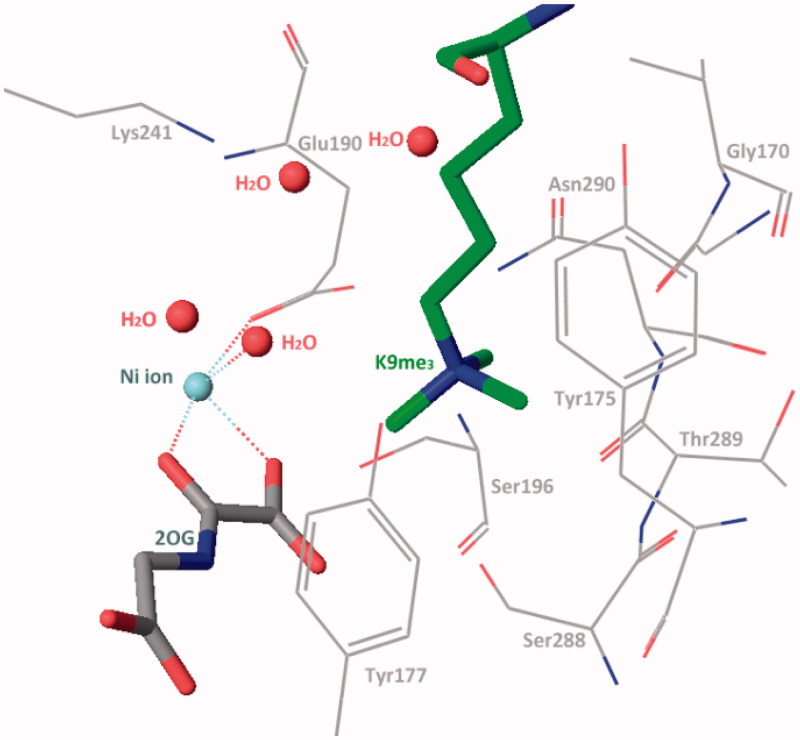
Trimethylated lysine side chain in catalytic region (PDB code: 2OQ6).

In the methylated lysine side chain binding sub-pocket, the positively charged nitrogen of methylated lysine side chain is surrounded by some nucleophilic side chains of Try177, Glu190, Ser288, and Asn290 (Figure 3). These three methyl groups project toward Asn290, Fe(II) ion (replace by Ni(II) ion in crystal structure), and Try177. At the one or two methylated state, the methyl group is preferentially pointing to Asn290 and then Try177. The rest positions are occupied by water molecules. This structural information elucidates the methylated state selectivity of KDM4A^73^.

KDM4A, together with 4B and 4C, can catalyze both H3K9me_3_ and H3K36me_3_ substrates, whereas the KDM4D could only recognize H3K9me_3_ motif. The main reason of the difference between KDM4s is a different recognition mode between H3His39 and Arg40 which is indicated by the crystal structure and some docking studies. The His90 and Leu75 in KDM4D present a steric clash with H3Arg40 compared with the corresponding residues Asn86 and Ile71 in KDM4A. The arginine in -1 position also plays an important role for both KDM4A and 4D: its side chain forms multiple hydrogen bonds with Asp135, Tyr175, and Glu169 in KDM4A, corresponding to Asp139, Tyr179, and Glu173 in KDM4D^75^.

**Figure 4.**
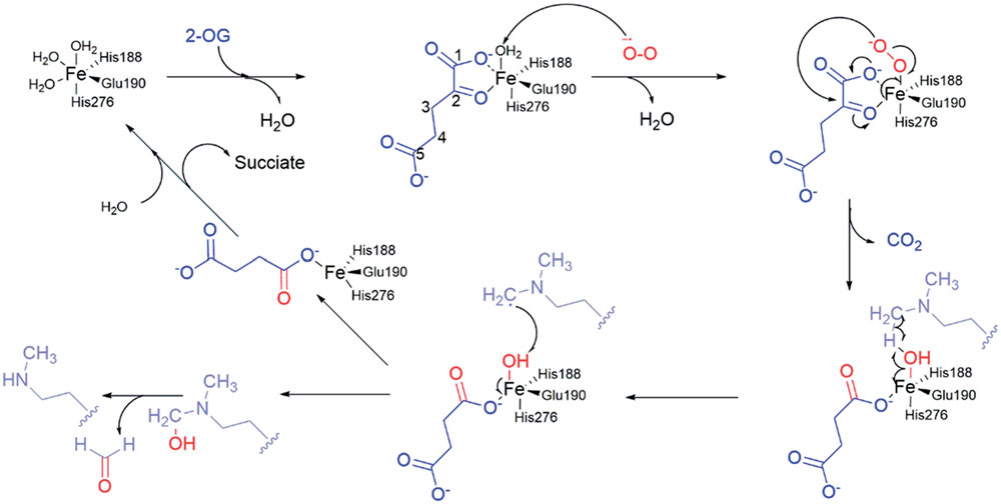
Mechanism of demethylation. 2-OG shows in blue; O2 shows in red; substrate lysine side chain shows in pink.

### Demethylation mechanism

Crystal-structural studies reveal the catalysis region of KDM4A and take insight of the catalysis process. Unlike the LSDs, JmjC KDMs can demethylate tri-, di-, and monomethylated modification in some specific sites. Furthermore, most JmjC KDMs are more inclined of tri- or di-methylated lysine. Their catalytic activity is dependent on Fe(II) ion and 2-OG. Its molecular mechanism has been well revealed^14^^,^^63^ (Figure 4). In the catalytic region of KDM4A, Fe(II) ion (replaced by Ni(II) ion) is chelated by residues His188, Glu190, and His276. Two water molecules are also involved in this site. In the co-crystal structure, 2-OG replaces the water molecules and interacts with the Fe(II) ion by its C1 carboxylate oxygen and the C2 ketone oxygen. The other carboxylate moiety of C5 forms hydrogen bonds with Tys132, Asn198, and Lys206 to stabilize the binding pose of 2-OG^71^. At the start, the water molecules are replaced by 2-OG (in blue) and then one molecule of negative oxygen ion (in red) attacks the Fe(II) positive ion. Then the negatively charged oxygen of the Fe(III)-O2 complex attacks the C2-position of 2-OG, resulting in decarboxylation to carbon dioxide, succinate, and Fe(IV) = O group. The Fe(IV) = O group’s oxygen forms a hydrogen bond with the hydrogen of the aminomethyl group of lysine side chain (in pink), which is nearby. The C–H bond of aminomethyl group breaks and then forms a carbon free radical. In the next step, the hydroxyl group transfers from the Fe(III) ion to the carbon free radical. The aminomethyl group is hydroxylated and then takes off one molecule of formaldehyde. At last, the succinate is replaced by water molecules or 2-OG. The 2-OG and succinate are also intermediate products in Krebs’s Cycle. So, it is presumed that this reaction could be influenced by energy metabolism.

## KDM4s inhibitors

**Figure 5.**
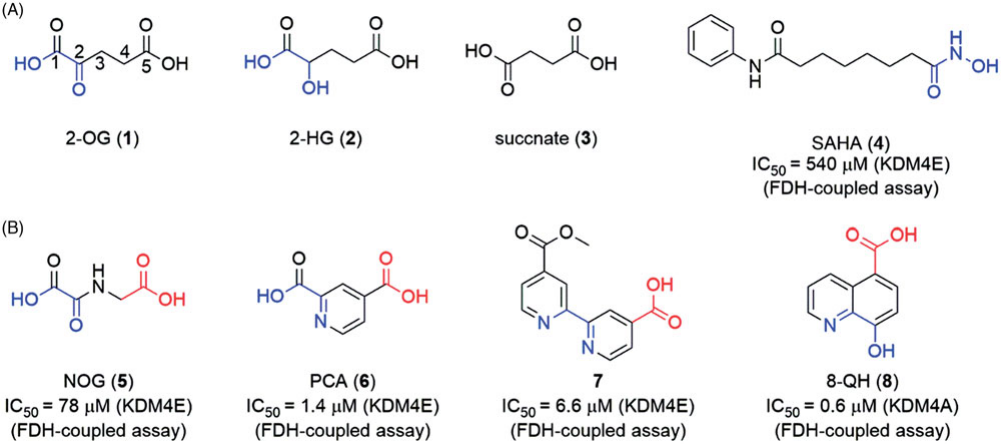
(A) Structures of endogenous substances and SAHA; (B) scaffolds of “generic” 2-OG-dependent inhibitor.

Like many other 2-OG-dependent enzymes, KDM4s are naturally inhibited by some 2-OG-like endogenous substances, such as alpha-hydroxygluarate (2-HG, **2**, the reduzate of 2-OG) and succinate (**3**, the by-product of histone demethylation)^76^^,^^77^ (Figure 5(A)). The 2-HG could be converted from 2OG and isocitrate by mutant isocitrate dehydrogenase (IDH) in some types of brain tumors^78^. The up-regulation of 2-HG increases the level of HIF1α by inhibiting PHD2 (a 2-OG-dependent oxygenase).

The development of JmjC KDM inhibitors was initiated after the identification of the enzymes. The primal drug discovery works focused on natural product and 2-OG mimics^79^. SAHA (**4**, an HDAC inhibitor with a metal-chelated moiety) also showed mild inhibitory activity. With a growing number of structural biology works being reported, SBDD strategy was used in the discovery of JmjC KDMs inhibitors. Several different chemotypes of inhibitors have been reported. Most of the inhibitors are 2-OG mimics with metal-chelated moiety which can bind to the Fe(II) ion in the catalytic core. Many natural inhibitors, such as catechol, also contain metal-binding moieties. Some other types of inhibitors (including Zn(II) ion ejection inhibitors, peptide based inhibitors, etc.) were also reported with limited quantity. So far, the 2-OG competitive inhibitors are still the major strategy for JmjC KDMs.

It should be indicated that it is still a challenge to improve selection among JmjC KDMs subfamilies. Inside KDM4s subfamily, no member-selective inhibitor has been reported as far as we know. Thus, the inhibitory activity on KDM4E (a pseudogene) or 4 C was sometimes used to represent the inhibitory activity of compounds on KDM4s. Additionally, the IC_50_ values of the various inhibitors were obtained by different methods, such as FDH-coupled assay, MS related assay, LANCE assay, and AlphaScreen. Although some reference compounds were involved in the experiments, a proper comparison of the activities of different compounds could be correctly done only within the context of the same assay. Thus, we label the types of test assay of the inhibitors’ IC_50_ values on the figures for reference.

### Metal-chelated inhibitors

As members of the 2-OG-dependent oxygenase family, JmjC KDMs can be inhibited by some 2-OG dependent enzymes inhibitors (**5–8**) which can coordinate the Fe(II) ion like 2-OG. These inhibitors are also called as “generic” inhibitors for 2-OG dependent oxygenases^24^^,^^79^ (Figure 5(B)). The common features of metal-chelated inhibitors are a 1,4-bidentate moiety (represented in blue in structures) and a carboxyl group (represented in red in structures). Based on different metal-chelated moieties, the inhibitors could be divided into (1) oxalyl acid scaffold; (2) hydroxamic acid scaffold; (3) hydrazide scaffold; (4) 8-hydroxygenquinoline (8-HQ) scaffold and benzimidazole pyrazolone scaffold inhibitors; (5) pyridine-based scaffold.

#### Oxalyl acid, hydroxamic acid, and hydrazide scaffold inhibitors

**Figure 6.**
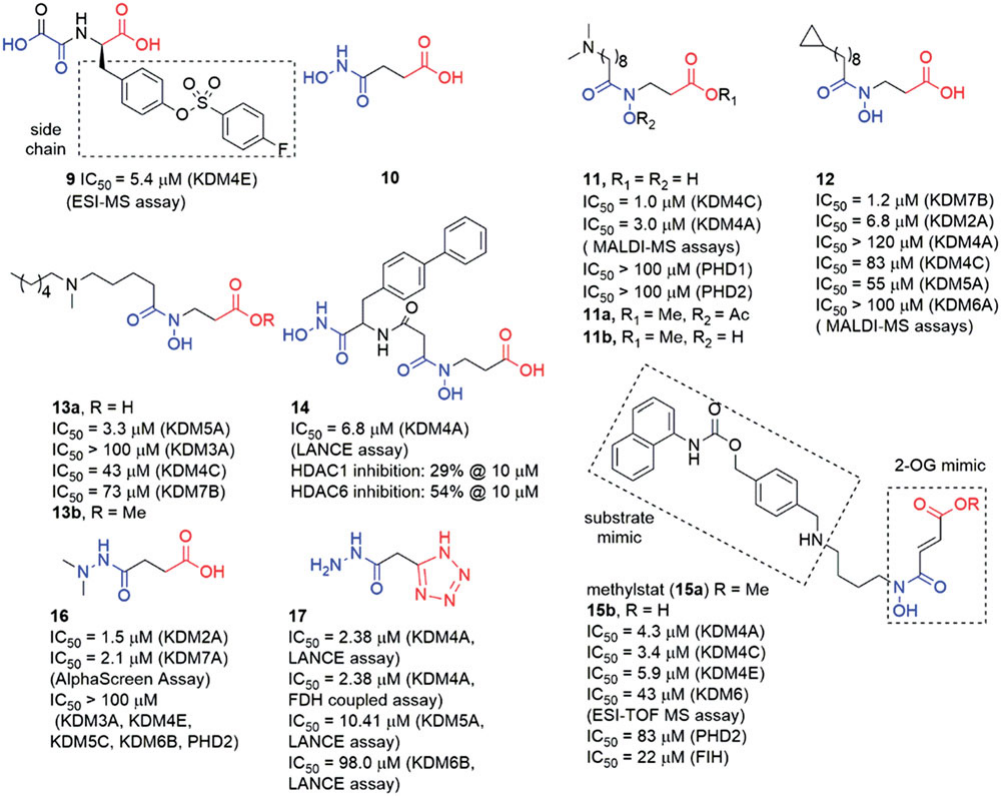
Structures of oxalyl acid, hydroxamic acid and hydrazide scaffold inhibitors.

*N*-Oxalylglycine (NOG, **5**) is a human HIF prolyl hydroxylase PHD2 inhibitor with *K*_i_ = 8 μM. As a 2-OG mimic, NOG showed a 24 μM IC_50_ value for KDM4A. Although the inhibitory activity of 5 is medium, its small molecular weight (MW) makes 5 a good hit for the development of KDM4s inhibitors^79^(Figure 6). Co-crystal structure indicates that the oxalyl group of NOG binds to the Fe(II) ion and the carboxyl group of glycine is positioned to form hydrogen bonds with Lys241 and Tyr177 of KDM4A^73^. The structural studies and modelling results imply that a subpocket is adjacent to the activity site of KDM4s but not exists in PHD2. Thus, one usual approach to provide selectivity toward KDM4A is to occupy this hydrophobic pocket by introducing a big side chain on NOG. By synthesizing and evaluating many N-substituted derivatives and amino acid derivatives of NOG, compound **9**, an N-oxalyl tyrosine derivate, was identified as a potent and selective KDM4A inhibitor. Its side chain occupies the large pocket beside the 2-OG^80^. Additionally, the different side chains of NOG can provide selectivity toward the subfamily of 2-OG dependent oxygenase^24^.

The metal-chelated moiety of SAHA, hydroxamic acid group (**10**), could replace the oxalyl group to bind the Fe(II) ion in KDM4s. Compound **11** was a hydroxamic acid derivative with a long carbon chain. The introduction of the long chain provided **11** good selectivity toward KDM4s over PHD2^81^. A combination of NCL-2 (a LSD1 selective inhibitor) with **11a** or **11b**, two prodrugs of **11**, displayed synergistic cell growth inhibitory activity on LNCaP, PC3, and HCT116 cell lines. However, all the three compounds did not show obvious inhibitory activity alone. Interestingly, replacing the dimethylamine moiety of **11** by a cyclopropyl moiety (**12**) led to good selectivity toward KDM2/7 subfamilies^82^. Docking study indicated that this selectivity was possibly due to the different environments around the subpocket of KDM4s and KDM2/7s: the pocket in KDM4A was composed of some polar residues such as Tyr179 and Asp137, while in KDM7A the residues belonged to some hydrophobic amino acid (Phe250, Phe359, and Tyr234). Moreover, **12** showed anti-proliferative activity in N2a, KYSE150, and Hela cell lines (GI_50_ value is 86, 16, and 40 µM, respectively) via downregulating E2F1 gene expression^83^.

Another hydroxamic acid derivative compound **13a** shared similar scaffold with **11** and **12**^84^. However, **13a** displayed selectivity toward KDM5A (IC_50_ = 3.3 μM) than KDM3A (IC_50_ > 100 μM), KDM4C (IC_50_ = 43 μM), and KDM7B (IC_50_ = 73 μM)^84^. The docking study showed that the interaction between the NH group and Asp412 in KDM5A might play an important role for the selectivity. Its methyl ester prodrug **13b** could increase the accumulated amount of H3K4me_3_ in a dose-dependent manner in A549 cell. In contrast, it had minor effect on the levels of H3K9me_3_ and H3K27me_2_. **13b** could also inhibit the proliferation of A549 cells.

Similar to SAHA, another HDAC inhibitor SW55 also showed weak inhibitory activity to JmjC KDMs with IC_50_ = 25.4 μM. Based on the scaffold of SW55, Jung et al.^85^ synthesized a series of 4-biphenylaline and 3-phenyltyrosine derived hydroxamic acids as KDM4A inhibitors. The best compound **14** contained two hydroxamic acid moieties and exhibited potent inhibitory activity to KDM4A with IC_50_ = 1.7 μM (LANCE assay). **14** also had potency for HDAC6 and HDAC1. In the proliferation experiments, **14** displayed antiproliferative effect against two human cancer cell lines: KYSE-150 and HL-60 with GI_50_ = 44 μM and 53 μM, respectively. An immunofluorescence microscopy assay ascertained that **14** could significantly increase the H3K9me_3_ in KYSE-150 cells.

The Methylstat (**15a**) is a selective inhibitor that links a 2-OG mimic moiety with a substrate mimic moiety from a HDAC inhibitor MS-275^86^. Compound **15a** showed good selectivity toward KDM4s than PHDs and HDACs, but it did not show potent inhibitory activity on KDM4s (IC_50_ = 4.3 μM). Compound **15a** inhibited KYSE150 cell growth with GI_50_ = 5.1 μM, but the unmethylated compound **15b** showed almost no effect on the cell growth.

The hydrazide group is not regarded as a good metal-chelated group because its amino nitrogen atom is potential to be protonated and lose its lone pair electrons. However, some simple hydrazide derivatives (**16** and **17**) displayed good JmjC KDMs inhibitory activity. Similar to **12**, the two amino methyl groups of **16** occupied the hydrophobic pocket of KDM7s to provide selectivity. In contrast, **17** is a selective KDM4s inhibitor with medium activity. The docking result indicated that the tetrazole group instead of the carboxylate interacted with KDM4s. Structure–activity relationship (SAR) studies indicated that the unsubstituted hydrazide group might be the reason for its selectivity.

#### 8-Hydroxygenquinoline (8-HQ) scaffold inhibitors

**Figure 7.**
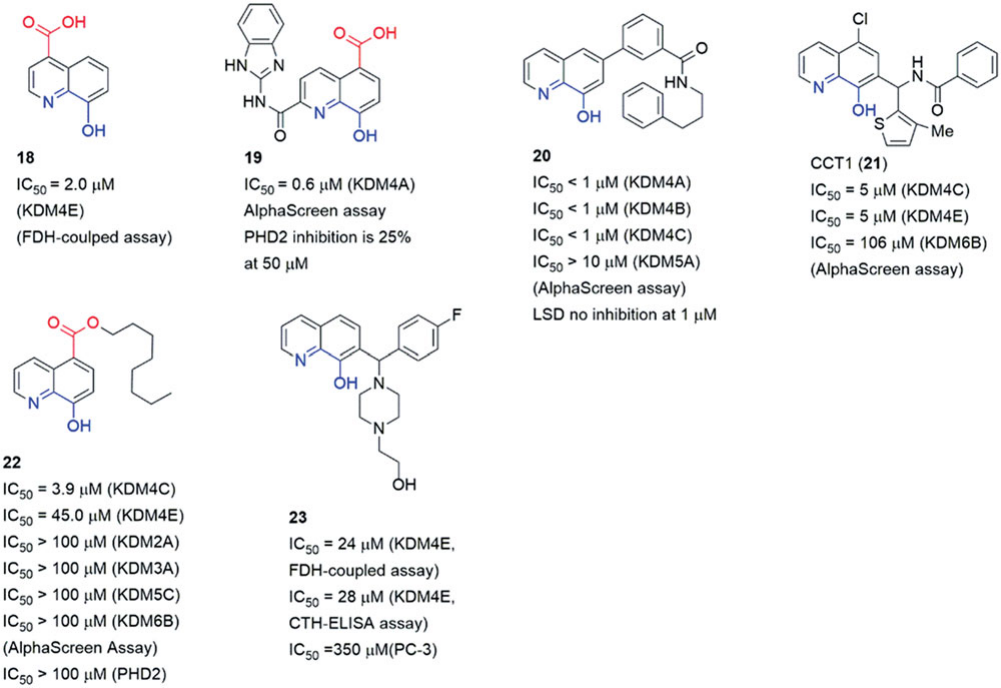
Structures of 8-hydroxygenquinoline core inhibitors.

8-HQ possesses potent coordinating ability and good metal-recognition property. A derivative of 8-HQ, 5-carboxy-8-hydroxyquinoline (**8**), is a potent KDMs inhibitor^87^. Notably, 4-carboxy-8-hydrogenquinoline (**18**) displays a better superimposition with 2-OG scaffold, but its activity is lower than the 5-carboxy derivative (Figure 7). Crystallographic studies demonstrated that **8** could cause translocation of the Fe(II) ion in activity site^88^.

Several 8-HQ derivatives were discovered from HTS assays or SBDD. Some derivatives were designed to enhance the selectivity towards KDM4s or the physicochemical property (**19–23**)^89–91^ (Figure 7). Such optimization enhances the selectivity of inhibitors on KDMs and antiproliferative activity on cancer cells. **19** displayed promising antiproliferative activities against three cell lines (HCT116, MCF-7, and A549) with IC_50_ = 1.5 μM, 27.6 μM and 21.7 μM. It also exhibited good aqueous solubility and appropriate Log*D*_7.4_, indicating a better physicochemical property than **8**^89^. Compound **20** was a selective KDM4s inhibitor and had high selectivity for a variety of cancer cells including PC3 cells that lack AR. It also inhibited the *in vivo* growth of tumours derived from PC3 cells and *ex vivo* human PCa explants. Additionally, **20** could block the binding of KDM4B to the promoter^90^.

Similar to the SAR of compounds **11** and **12**, the length of the carbon chain of compound **22** offered selectivity among subfamilies of JmjC KDMs^92^. The carbon chain also provided a better cell permeability. Treatment with **8** or its ester derivative **22** caused a dose-dependent increase in H3K9me_3_ fluorescence intensity, implying KDM4A inhibition in Hela cells.

Compound **23** (as known as CBN207192) indicated modest inhibitory activity and promising selectivity toward KDM4s by employing the formaldehyde dehydrogenase (FDH)-coupled fluorescence assay and the CTH-ELISA (IC_50_ = 24 μM, FDH-coupled fluorescence assay and IC_50_ = 28 μM, CTH-ELISA). All members of the KDM4(A-E) subfamily were inhibited with similar potencies^93^. Cell-based activities assays demonstrated its anti-cancer effect at high inhibition concentrations by reducing the proliferation of PCa cell lines.

#### Pyridine-based inhibitors

**Figure 8.**
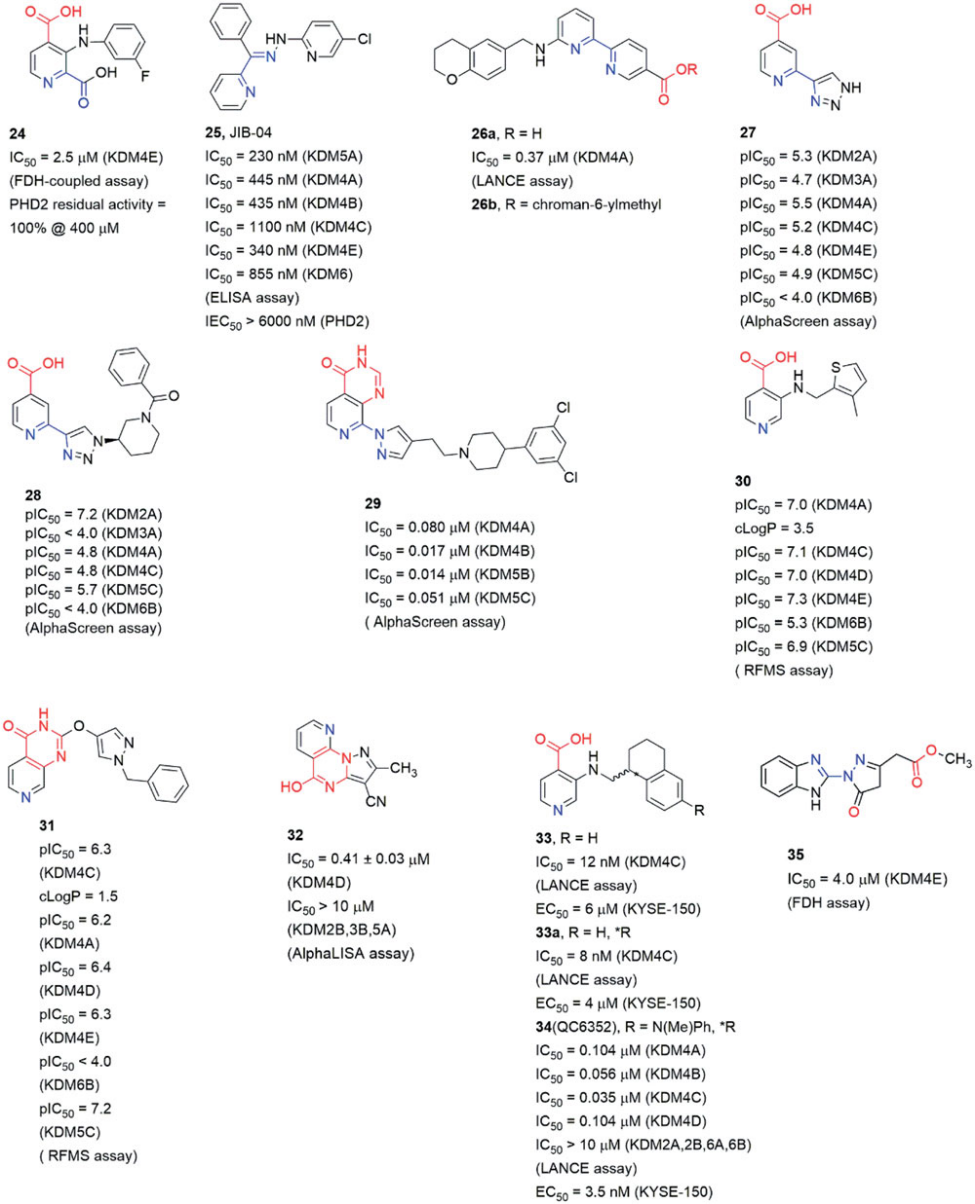
Structures of pyridine-based inhibitors.

Pyridine-based inhibitor 2,4-pyridinedicarboxylic acid (**6,** PCA) is one of the most potent inhibitors for KDMs, although its physicochemical properties and selectivity are not satisfactory. However, the pyridine moiety as metal ion binding group is often involved in KDM4A inhibitor scaffolds^79^ (Figure 8). The pyridine nitrogen and the 2-position carboxylate of PCA bind to the Fe(II) ion in a bidentate manner as the oxalyl acid group of NOG. And the 4-position carboxylate forms hydrogen bonds with Lys241 and Tyr177. Introducing a phenyl ring on 3-position to occupy the pocket beside activity site, **24** displayed selectivity toward KDMs than PHD2^94^.

Based on a cell-based assay (named locus derepression), JIB-04 (**25**) was identified as a JmjC KDMs inhibitor^95^. Compound **25** showed a pan inhibitory activity on JmjC KDMs but weak affection on PHD2. It was noteworthy that the Z-isomer which lacked a pyridine-based chelated moiety showed almost no activity. It indicated that **25** may inhibit KDM4A in a Fe(II) ion binding manner. Compound **25** inhibited KDM4A activity *in vivo* and its action was modulated by KDM4A enzyme level. Moreover, in an immune-competent tumour mouse model, **25** diminished tumour growth compared with vehicle-treated mice.

Compound **26a** was identified by Jung *et al.* through virtual screening and structural optimizing as a potent KDM4A inhibitor with IC_50_ = 0.94 μM^96^. This inhibitor also showed good potency towards KDM5A (IC_50_ = 0.44 μM) and moderate potency towards KDM6B (IC_50_ = 36.5 μM). The co-crystal structure of KDM4A and **26a** indicated that the nitrogen atoms of pyridine and pyrimidine chelated the Fe(II) ion while the carboxylate group interacted with Lys206 and Tyr132. The secondary amine formed a hydrogen bond with the carboxylate of Glu190. And the terminal pyridine ring protruded into a large pocket formed by Tyr175, Val171 and Asp191. The combination of optimal side chain in **26a** with this ester led to **26b**, which was indeed soluble at much higher concentrations and exhibited obvious inhibition on proliferation of KYSE-150 cells.

Brennan et al. replaced carboxylate group by a triazole moiety to gain compound **27**. Compound **27** showed moderate inhibitory activity toward KDM4s (pIC_50_ = 5.5 ± 0.33)^97^. Co-crystal structure of KDM4A and **27** indicates that some five-member sp^2^-hybridized nitrogen containing heterocyclic could play the same role of pyridine in **7** or carboxylate in **6**. Moreover, introducing some moieties on the heterocyclic can provide more selective and potent inhibitors. A number of analogous of **27** were synthesized leading to selective KDM inhibitors. One of the analogous, compound **28**, was a potent and selective KDM2A inhibitors with pIC_50_ = 7.2 (pIC_50_ = 4.8 for KDM4).

Recently, another co-2-OG and -substrate mimic **29** was reported. Although the design of the inhibitors started from a 2-OG mimic from an HTS assay, the docking result showed that the chlorobenzyl piperidine group inserted into the substrate methylated lysine side subpocket^98^. This may be the reason for its good potency on KDM4s (IC_50_ value is 17 nM for KDM4B, and 80 nM for KDM4A). In this scaffold, the 4-carboxylate group was replaced by pyrido[3,4-d]pyrimidin-4(3*H*)-one group (in red). This replacement led to an obvious enhancement of cellular permeability.

The pyrido[3,4-d]pyrimidin-4(3H)-one group was developed by Westaway et al.^99^^,^^100^. In Westaway’s work, some 3-amino-4-pyridine carboxylate derivatives have been identified as hits for KDM4 inhibitors. Compounds **30** and **31** were developed as the potent cell penetrant inhibitors with IC_50_ ≤ 100 nM^99^. These inhibitors bound Fe(II) ion only via the nitrogen of pyridine ring (single pyridine binding JmjC KDMs inhibitors was common in patent literatures). In a subsequent work, the 4-carboylate group and the amino group were linked to form a bicyclic scaffold which kept potency^100^. This replacement abandoned the carboxylate group which frequently occurred in KDM4s inhibitors and it may be able to be introduced into some other types of inhibitors to optimize the physicochemical properties.

In Zhen Fang’s work^101^, a novel selective KDM4D inhibitor (**32**), which had a pyrazolo-[1,5-a]-pyrimidine-3-carbonitrile scaffold similar to **31**, was reported. A molecular docking-based virtual screening against various chemical libraries include Specs and ChemDiv was performed to retrieve two compounds with modest inhibition rate against KDM4D (> 50% at 10 μM). The one with better selectivity against KDM4D became the starting point of structural optimization which led to compound **32** and analogs. Compound **32** was the most potent one with an IC_50_ of 0.41 ± 0.03 μM and displayed good selectivity for KDM4D over other tested KDM family members (> 10 μM against KDM2B, 3B and 5A).

Chen et al. developed a novel series of potent KDM4 inhibitors with promising selectivity from a small fragment lead using structure-based design. The SAR study led to the most potent and efficacious inhibitor compound **34** (as known as QC6352)^102^. They explored the substitutions of the 3-aminoisonicotinic acid nitrogen then identified the tetrahydronaphthalene compound **33** as a potent inhibitor of KDM4C (IC_50_ = 12 nM). Compound **33** also demonstrated a measurable inhibition activity to cell proliferation in the KYSE-150 cell line (EC_50_ = 6 μM). The *R* enantiomer, **33a**, was determined to be the active enantiomer (KDM4C IC_50_ = 8 nM, KYSE-150 cell EC_50_ = 4 μM). After Molecular modelling of **33a**, researchers developed a series of derivatives with 6-position substitutions which were designed to impart favourable hydrophobic interactions and improve cellular permeability. Among them all, compound **34** showed favourable *in vivo* efficacy (KYSE-150 cell EC_50_ = 3.5 nM, tumour growth inhibitions =61% at dose level:50 mg/kg) in a breast cancer PDX model and reduced the tumour initiating cell populations, which were associated with resistance to chemotherapy treatments.

In a recent work, Metzger et al. established a method to isolate and propagate breast cancer stem-like cells (BCSC) from individual triple-negative tumours stemmed from patients after neoadjuvant chemotherapy. This method is intended to assess the antitumor properties of **34** (QC6352)^103^. After they validated KDM4A is directly related to proliferation and xenogaraft tumour growth of BCSC, **34** was tested if it is qualified for the treatment of BCSC-originating tumours. Results showed that **34** revealed excellent potency and selectivity against the KDM4A-D subfamily (hundredfold selectivity over KDM2 and KDM6 and weak inhibition of KDM5B), with some favourable pharmacokinetic properties. Moreover, it’s corroborated that **34** blocked proliferation and self-renewal of BCSCs through EGFR regulation *in vitro*, and xenograft tumour growth of BCSCs *in vivo*.

David et al. who identified the **23** also validated compound **35** (CBN209350) as a novel benzimidazole byrazolone scaffold KDM4 inhibitor by HTS. Compound **35** represented an undescribed scaffold in KDM inhibitors, it exhibited some specificity toward KDM4 enzymes. Compound **35** also displayed cytostatic activity in PCa cell lines at a higher concentration. By Rosen et al.^94^, they described that **35** had key structural similarities to other privileged scaffolds that inhibited Fe^2+^/α-KG-dependent hypoxia inducible factor prolyl hydroxylase (PHD) enzymes such as the Molidustat.

**Figure 9.**
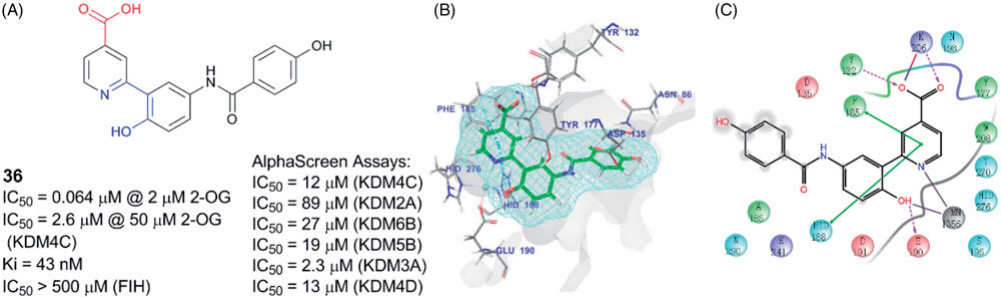
Compound **36** and its binding pose. (A) Structure of **36**; (B) and (C) **36** binding pose (PDB code: 5A7W).

A docking and linking of fragment strategy was applied to discover novel, potent JmjC KDM inhibitors^104^. After analyzing the docking models of two fragments, 5-carboxypyridine and 5-aminosalicylate, a new fusional structure type was designed and optimized to get **36** (Figure 9). This combined approach improved affinity by ∼3 log-orders (*K*_i_ = 43 nM). This compound showed great potency with an IC_50_ value of 64 nM (at 2 μM of 2-OG) and good selectivity towards KDM4s than PHD2. Unlike most of Fe(II) ion coordinating inhibitors, **36** was a 1,5-bidentate ligand instead of a 1,4-bidentate ligand. Co-crystal structure demonstrates that the pyridine ring and benzene ring form dihedral angle of 36°. By testing its KDM4C inhibitory activity in various concentrations of 2-OG or substrate peptide, **36** showed competition with 2-OG but no competition with the substrate peptide (IC_50_ = 4.8 μM at 50 μM of 2-OG and 50 μM peptide, IC_50_ = 5.5 μM at 50 μM of 2-OG and 250 μM peptide, IC_50_ = 1.1 μM at 5 μM of 2-OG and 50 μM peptide).

### Non-chelated inhibitors

**Figure 10.**
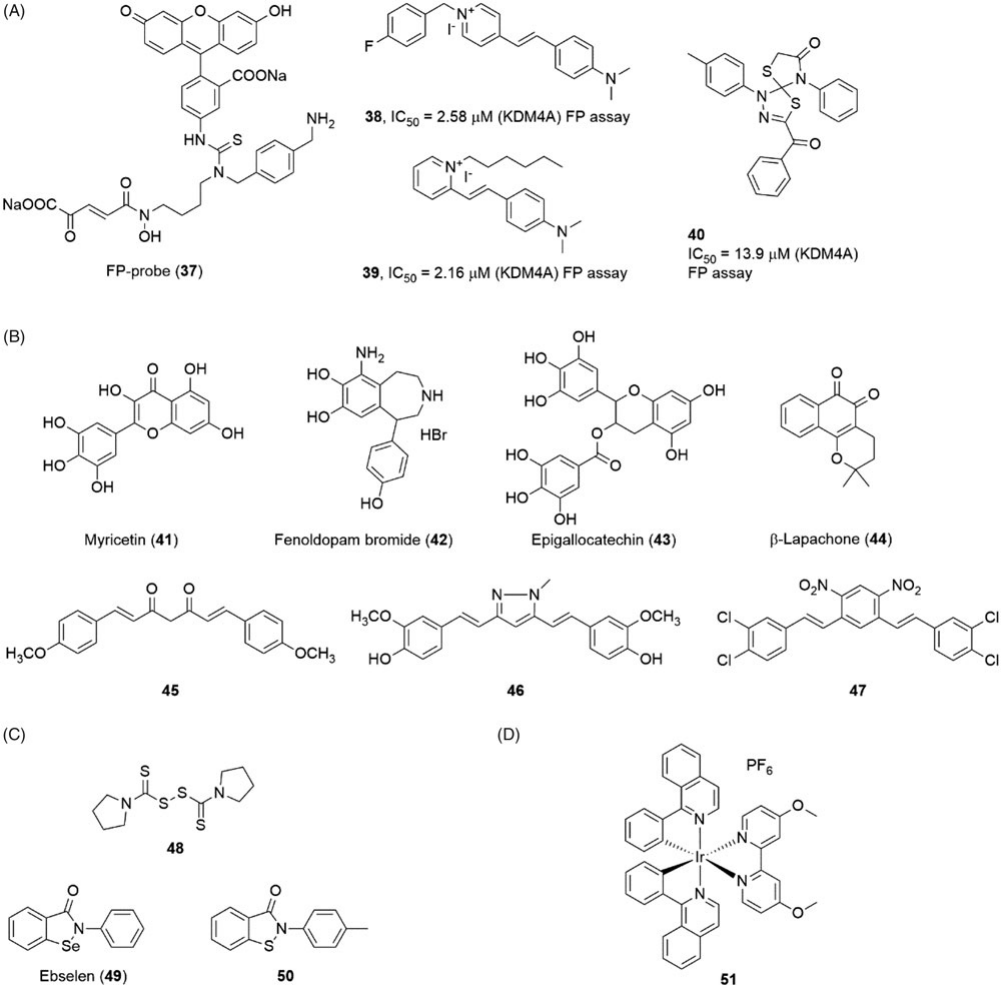
Structures of (A) FP-probe and screen inhibitors; (B), natural product inhibitors; (C) Zinc ion ejection inhibitors; and (D) metal-containing inhibitor.

The metal-chelated agents reveal good potency on KDM4s. But considering the large number of metal-containing proteins in cells, this feature also rises the potential off-target risk of the inhibitors. The non-chelated inhibitors are also necessary for the KDM4s. Linking a fluorescent group FITC (fluoresceinisothiocyanate) with a KDM4s inhibitor resulted in a FP-Probe(**37**) which was used in fluorescent polarization (FP) assay to screen novel JmjC KDMs inhibitors^105^ (Figure 10(A)). Using this fluorescent probe, several non-chelated inhibitors, compounds **38**, **39** and **40**, were identified through a FP-based HTS assay^106^. Compounds **38** and **39** shared a pyridinium fragment and a dimethylamino-styrene fragment. All three compounds had moderate inhibitory activities in FP assay with IC_50_ = 2.58 μM, 2.16 μM, and 13.9 μM.

### Natural originated inhibitors

Many natural products show KDM4s inhibitory activity (compounds **41**, **43** and **44**) (Figure 10(B)). Although a large part of them contain metal-chelated groups (such as catechol scaffold **41**, **42** and **43**), we prefer to discuss the natural products separately. Many catechols (including some synthesis compounds such as Fenoldopam **42**) showed inhibitory activity to KDM4E with moderate IC_50_ value (1–10 μM)^107^. However, a shape and electrostatic similarity search indicated that the simple catechol moiety could inhibit KDM4A alone. The docking model and SAR study both indicated that the two adjacent hydroxide groups chelated the Fe(II) ion and the electrical property of the phenyl ring greatly affected the affinity^108^. This study implied that natural catechol may be too complex for KDM4s. And on the other side, the ultra-small molecule inhibitors might be an opportunity for KDM4s. Curcumin and its derivatives (**45**, **46**, and **47**) also showed good activity on inhibiting histone demethylation in cell level^109^^,^^19^. However, the curcumin is a well-known pan-targets inhibitor and is regarded as one of the pan-assay interference compounds (PAINS)^110^.

### Zinc ion ejection inhibitors

Ejection of structural Zn(II) ion is another possible strategy for inhibiting KDM4A. Structural study revealed that the Zn(II) ion binding site is very close to the catalysis region in KDM4A^73^. The Zn(II) ion binding motif (His220, Cys234, Cys306 and Cys308 in KDM4A) was next to the substrate-recognition residues, Lys241 and Arg309. So, this motif may play an important role for keeping substrate-recognition conformation. Several Zn-ejecting compounds, including disulphiram (**48**) and ebselen (**49**), could inhibit KDM4A with IC_50_ = 3.3 and 10.6 μM, respectively^111^(Figure 10(C)). However, considering Zinc ion is contained in a large number of proteins, the selectivity of Zinc ion ejection inhibitors should be further studied. An ebselen-structure similar compound **50** was identified as a KDM5B inhibitor through HTS assay. Compound **50** also showed a moderate activity for KDM4E with IC_50_ = 28 µM).

### Metal-contain inhibitors

Nickel ion, together with some other divalent ion like Co(II), could inhibit JmjC KDMs activity^112^. The IC_50_ value of nickel ion is 25 μM for KDM3A. These ions could replace the Fe(II) ion in the catalytic site to block the hydroxylation process. Recently, an Iridium (III) complex **51** was reported as a KDM4 inhibitor with IC_50_ = 15 μM^113^(Figure 10(D)). Absorbance spectrum and ESI mass spectrometry showed that **51** did not inhibit KDM4A via ion-sequestration method. In the A549 cell line, **51** led to the increase of H3K9me_3_ and the amplification of the p21 gene promoter. Moreover, **51** displayed weaker potencies on KDM5A and KDM3 than KDM4 in an ELISA assay which was based on H3K4me_3_ and H3K27me_3_ antibody separately.

### Peptide-based inhibitors

**Figure 11.**
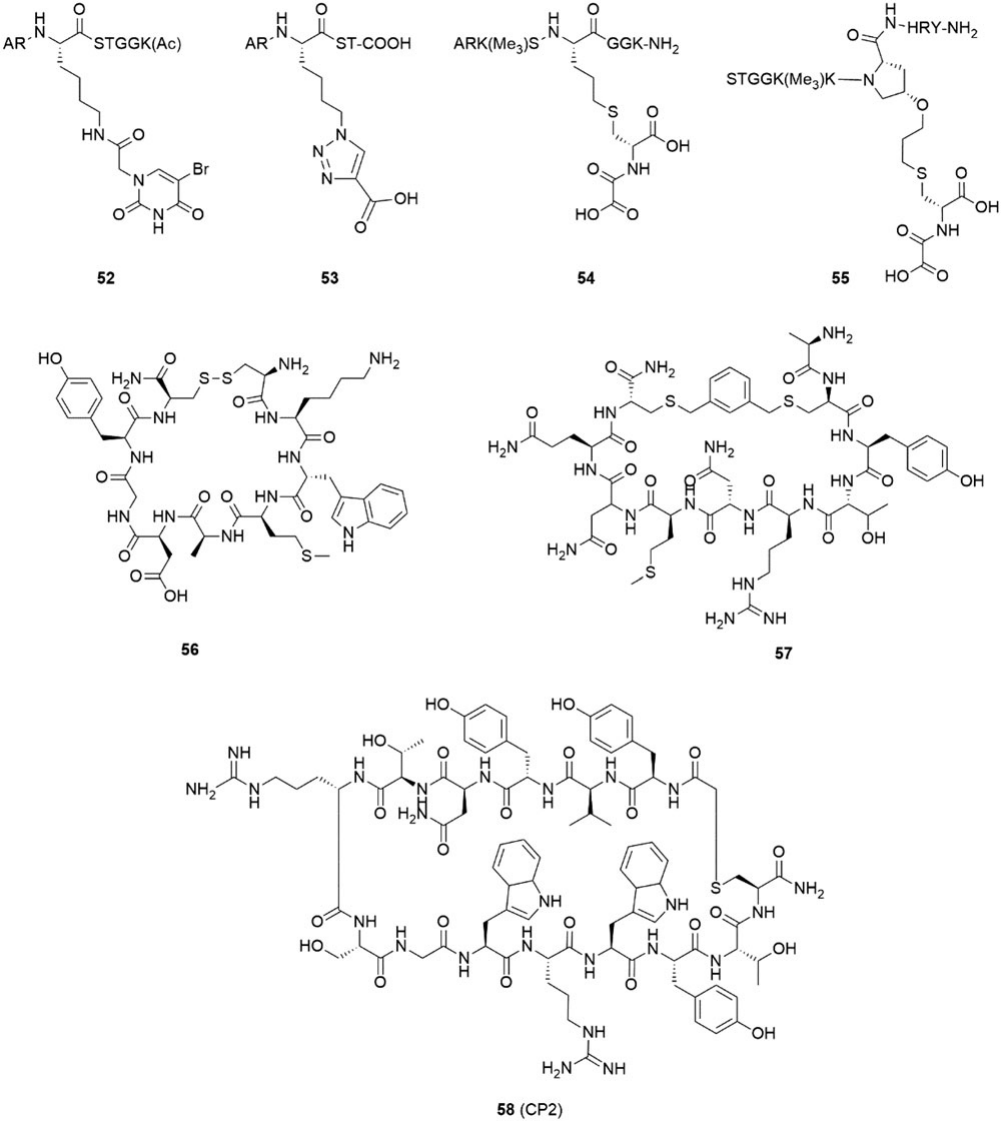
Peptide based inhibitors.

Some attempts for developing substrate mimic inhibitors were truncating or modifying substrate peptides (Figure 11). By truncating the H3K9me_3_ substrate, H3(7–14)Kme_3_ peptide was identified and displayed the best KDM4A affinity (*k*_cat_ = 0.01 min^−1^, *K*_M_ = 121 μM), even better than some longer peptides^114^. In accordance with previous structural study, the absences of Gly–Gly led to a rapid decreasing in enzyme kinetic activity (*k*_cat_ value) for KDM4C. Introducing a metal-chelated moiety (uracil) or a carboxylate group compounds **52** and **53** which showed moderate inhibition activities to KDM4A^114^. Compounds **54** and **55** were obtained by linking NOG (*N*-oxalylcysteine) with substrate peptides^115^. The two inhibitors showed better inhibitory activities and provided selectivity between KDM4A and 4 D. However, **54** and **55** did not link NOG to the lys9 side chain but the 10 position of H3K9me_3_ peptide or the Pro38 of H3K36me_3_ peptide.

Two peptide inhibitors were developed through a hydrogen/deuterium exchange coupled to mass spectrometry (HDX-MS) method^116^. After amino acid replacement, truncation, and chemical modification, two cyclic peptides (**56** and **57**) showed KDM4C inhibitory activities. It was important that the two peptides inhibited the KDM4C in a substrate- and 2-OG-independent manner. Additionally, the two peptide inhibitors bound to KDM4C in different regions. This study may provide some novel binding sites for KDM4C inhibitors.

Some highly potent and selective cyclic peptide inhibitors of KDM4A-C were developed by Random nonstandard Peptides Integrated Discovery (RaPID) system using two thioether-macrocycle libraries^117^. Compound **58** (CP2) demonstrated potent IC_50_ against KDM4A-C (IC_50_ = 42 nM, 33 nM, and 39 nM against KDM4A, B, and C, respectively) in a luminescence-based AlphaScreen assay. Moreover, **58** represented an exceptional selectivity in intra-subfamily: it displayed high potency against KDM4A-C and much less active against KDM4D-E (IC_50_ = 6270 nM, 9200 nM against KDM4D, KDM4E, respectively). Arg6 of **58** showed a significant function for KDM4A binding which indicated different binding modes between histone substrates (H3K9me_3_/K36me_3_) and **58**. Compound **58** derivatives based on structure-guided and mass spectrometry (MS)-guided modifications revealed meliorative stability in intracellular target. Although further studies of cyclic peptides at cellular level are necessary for deeper investigation.

## Other JmjC KDM inhibitors

**Figure 12.**
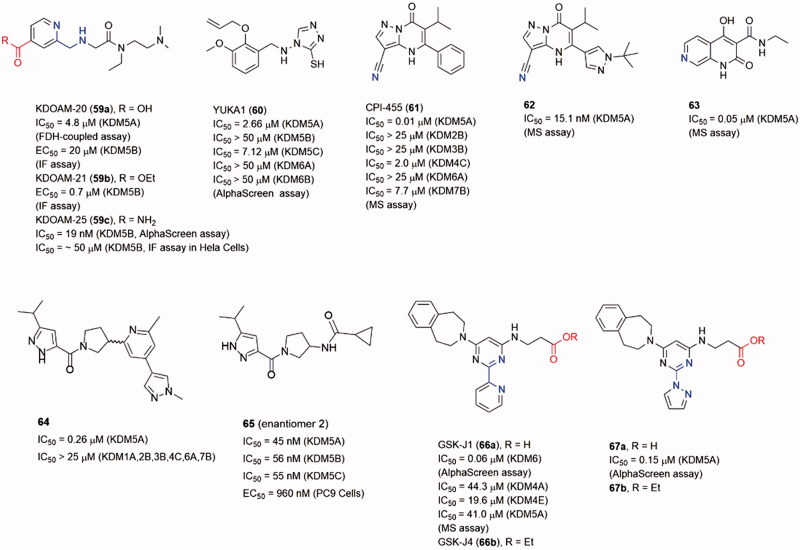
Structures of some other subfamily selective inhibitor.

Selective inhibitors for JmjC KDMs subfamilies are important as chemical biological tools to explore the biological roles of the JmjC KDMs^17^^,^^28^^,^^118^. Although discovery of the selective inhibitors is difficult, several selective inhibitors have been reported as mentioned before (such as **12** and **16** as KDM7 inhibitors, **28** as KDM2 inhibitor). To make an integrated comprehension for JmjC KDM inhibitors, several other subfamily selective inhibitors will be introduced in this section (Figure 12).

### KDM5 subfamily inhibitors

After KDM4 subfamily, the roles of KDM5 subfamily in cancer have attracted increasingly attention recently^119^. Especially, KDM5A and 5B could promote the development of drug tolerance and maintain tumour-initiating cells. Many evidences indicate that KDM5s are potential targets for cancer therapy. Meanwhile, many KDM5 selective inhibitors have been disclosed as anticancer agents.

Compound **59a** (KDOAM-20), and its cell-permeable ethyl ester derivative **59b** (KDOAM-21), were potent and selective KDM5s inhibitors^120^^, ^^121^. Several structural studies based on **59a** and its derivatives revealed the details of its binding pose in KDM5A. Treatment of MCF7 and MDA-MB-231 breast cancer cells with **59b** significantly increased the global levels of H3K4me_3_ while had little impact on H3K4me_2/1_ or other histone methylated marks. In colony-formation experiment, the growth of MCF7, BT474, and ZR-75–1 cells was inhibited at 5 μM of **59b**. Treatment of Hela cells transiently overexpressing WT KDM5B with **59a** and **59b**, assessed by immunofluorescence (IF) assay, showed potent inhibition of H3K4me3 demethylation, with EC_50_ = 20 μM and 0.7 μM, respectively. Unexpectedly, **59b** still showed modest activity against KDM5B in cells despite it has low cell permeability^122^.

In Tumber’s work^123^, researchers replaced the ester of KDOAM-21 with a less labile group to improve KDM5 potency and selectivity. Some amide derivatives were synthesized, among them **59c** (KDOAMA-25) showed the highest potency, with a KDM5B IC_50_ of 19 nM. Through AlphaScreen or MALDI-TOF enzymatic assays, **59c** was proved to be selective for KDM5 sub-family, because the IC50 values for other 2-OG oxygenases were over 4.8 μM. The cellular activity of **59c** was assessed in an IF assay by using an overexpression system in HeLa cells, it can increase H34Kme_3_ levels in a two-digit micromolar range. Compound **59c** also showed an inverse relationship between KDM5B expression and overall survival from three clinical multiple myeloma trials’ data, suggesting a potential application for KDM5B inhibitors in myeloma therapy.

Yan et al.^124^ identified some small molecule inhibitors through a HTS assay of full-length KDM5A^125^. YUKA1 (**60**) was one of the potent inhibitors with IC_50_ = 2.66 μM for KDM5A and was a cell permeable compound. But it showed little to no activity against KDM5B, KDM6A, and KDM6B. Treatment with **60** led to increased H3K4me_3_ levels and inhibited growth of Hela and ZR-75–1 cells which depended on KDM5A expression for their proliferation. In contrast, it exhibited no significant effect to MCF7, MCF10A, and PC9 cells. As KDM5A was showed to mediate drug tolerance, YUKA1 displayed the ability to prevent drug tolerance in EGFR-mutant lung cancer cells treated with gefitinib and HER^2+^ breast cancer cell treated with trastuzumab.

CPI-455 (**61**) was identified as an inhibitor of KDM5 through an HTS assay against KDM4C JmjC domain and subsequent structural modification work^124^^,^^125^. It exhibited good potency toward KDM5A with IC_50_ = 0.02 μM and KDM4C with IC_50_ = 0.62 μM. **61** could specifically increase H3K4me_3/2_ in a dose-dependent manner in PC9, M14 and SKBR3 cells. In the co-crystal structure, the nitrile N atom of **61** interacted with the active Fe(II) ion; the carbonyl O atom formed hydrogen bonds with Lys501 and Asn575 (PDB code 5CEH). Treatment with **61** reduced the number of surviving cells after lethal drug exposures in some cell culture models. Further lead optimization work led to a potent, orally bioavailable KDM5 inhibitors **62**^126^. Compound **62** did not display improved potency against **61** (IC_50_ = 15.1 versus 10.0 nM in parallel experiment), but it possessed a more “balanced” human plasma protein binding (hPPB) and cell permeability which led its superior cell potency. This inhibitor also exhibited an excellent pharmacokinetic (PK) profile in mice. A hybrid naphthyridone inhibitor **63** that combined **61** and another pyrido[3,4-d]pyrimidin-4(3*H*)-one KDM5 inhibitor also displayed good potency (IC_50_ = 0.05 μM for KDM5A) *in vitro*^127^.

In Liang’s work^128^, some KDM5 inhibitors with potent, selective and orally bioavailable characteristics were identified through a high-throughput screening (HTS) of the Genentech/Roche library. From the HTS campaign, **64** (as a racemic mixture) had reasonable physiochemical properties, excellent KDM5 potency and selectivity (IC_50_ = 0.26 μM against KDM5A). After comprehensive SAR studies and optimizations, a more potent compound **65** (IC_50_ of 45, 56, and 55 nM against KDM5A, KDM5B and KDM5C, respectively; EC_50_ = 960 nM in PC9 cells), was identified with promising *in vivo* PK profiles.

### KDM6 selective inhibitor

Members of KDM6 (JmjD3) subfamily can demethylated the H3K27me_3_ mark. KDM6s are involved in several physiological functions, including the inflammatory response. GSK-J1 (**66a**) was a selective KDM6 inhibitor with IC_50_ = 60 nM in AlphaScreen assay^129^. In the co-crystal structure, the pyridyl-pyrimidine biaryl made a bidentate interaction with the catalytic metal; the propanoic acid of **66a** mimicked 2-OG binding by Lys1381, Thr1387 and Asn1480; the aromatic ring of the tetrahydrobenzazepine moiety sat in a narrow cleft between Arg1246 and Pro1338. The ethyl ester prodrug **66b** was examined for the efficacy at inhibiting the LPS-induced response of human primary macrophages derived from healthy volunteers. Administration of **66b** significantly reduced the expression of 16 in 34 LPS-driven cytokines by PCR array. Xiong et al. designed and synthesized a series of derivatives of **66a** to study the detail of SAR of this scaffold of selective KDM6 inhibitors^130^. The representative compound **67a** had an equivalent potency against **66a** (IC_50_ = 0.15 μM versus 0.15 μM). However, its ethyl ester prodrug **67b** had a higher activity for inhibition of TNF-α production in LPS-induced murine macrophage cell line Raw264.7 cells.

## Future preview

Histone modifications are basic and vital post-transcription regulations for normal and tumor cells in epigenetic process. Histone lysine methylation seems a “massage-contain” step in the whole epigenetic regulation. A big number of methyltransferases and demethylases work together and elaborately control the behaviour of DNA and chromatin. It seems very hard to figure out the relationship among the histone modifications and even just among the KDMs. Some scholars raised the questions^131^: was histone methylation a “cause or cog” in epigenetics? Would the affection of blocking single KDMs be drowned in total epigenetic regulatory flood? Further research works need to carry on for these questions.

However, overexpression of some histone modification enzymes, such as G9a, LSD1, MLL1, is certain to contribute to cancers^2^^,^^3^. KDM4 is involved in tumourigenesis in different types of cancers. Biological and physiological studies demonstrate that KDM4 is a promising target for cancer, especially breast and prostate cancer. Thus, the KDM4 inhibitors are still worth being expected as a strategy for cancer^21^.

Moreover, targeted indication of JmjC KDMs inhibitors should not be limited in cancer treatment. As a milder method to affect gene transcription, histone methylation and demethylation could be involved in many therapeutic strategies for some other conditions. For example, some selective H3K27 demethylase KDM6 inhibitors showed anti-inflammation activity^129^.

The development of KDM4s inhibitors is a tough but attractive work for improvements of potency and selectivity. Recently, some breakthroughs were reported^98–100^^,^^104^^,^^120^. We note that both academia and industry are participating in this work^132^. Several future directions of KDM4s inhibitors may be focused on (a) more potent KDM4s inhibitors are still in urgent need, very limited number of nanomole level inhibitors have been presented now; (b) JmjC KDMs subfamily-selective inhibitors should be designed and synthesized for clinical application and providing biological chemistry tools; (c) some other non-2-OG mimic inhibitors are very desirable while a number of 2-OG-dependent enzymes, such as PHD2, are potential off-targets; (d) some further breakthroughs of the inhibitors may focus on physicochemical and pharmacokinetics property.
